# The Sensitivity Prediction of Neoadjuvant Chemotherapy for Gastric Cancer

**DOI:** 10.3389/fonc.2021.641304

**Published:** 2021-04-16

**Authors:** Juan Sun, Xianze Wang, Zimu Zhang, Ziyang Zeng, Siwen Ouyang, Weiming Kang

**Affiliations:** ^1^ Chinese Academy of Medical Sciences and Peking Union Medical College, Beijing, China; ^2^ Peking Union Medical College Hospital (CAMS), Beijing, China; ^3^ Department of General Surgery, Peking Union Medical College Hospital (CAMS), Beijing, China

**Keywords:** gastric cancer, biomarkers, inflammatory indicators, imageological assessments, neoadjuvant chemotherapy

## Abstract

The overall efficacy of neoadjuvant chemoradiotherapy (NACT) for locally advanced gastric cancer (LAGC) has been recognized. However, the response rate of NACT is limited due to tumor heterogeneity. For patients who are resistant to NACT, not only the operation timing will be postponed, patients will also suffer from the side effects of it. Thus, it is important to develop a comprehensive strategy and screen out patients who may be sensitive to NACT. This article summarizes the related research progress on the sensitivity prediction of NACT for GC in the following aspects: microRNAs, metabolic enzymes, exosomes, other biomarkers; inflammatory indicators, and imageological assessments. The results showed that there were many studies on biomarkers, but no unified conclusion has been drawn. The inflammatory indicators are related to the survival and prognosis of patients under NACT. For imageological assessments such as CT, MRI, and PET, with careful integration and optimization, they will have unique advantages in early screening for patients who are sensitive to NACT.

## Introduction

Gastric cancer (GC) is one of the most common digestive system tumors. According to GLOBOCAN estimates in 2018, the prevalence of GC ranks the fifth among all cancers, with approximately 1.034 million new cases worldwide. Meanwhile, GC-related deaths rank the third, with more than 783,000 patients annually (1). In China, there were 679,000 new cases and 498,000 deaths in 2015, which accounted for 42.6 and 45% of the global total, respectively (2).

Since early-stage GCs are absent of specific symptoms, 80–90% of GC patients are in the advanced stages at their first visits (3, 4), and the proportion of patients with stage II–III GCs in China is as high as 58.0% (5). Surgery is the main treatment for GC, but the long-term survival rate of LAGC patients after surgery is still less than 20–30% (4). Therefore, the NACT which aims at improving the prognosis of LAGC patients came into being. The superiorities of NACT, such as reducing the tumor size, achieving the complete pathological remission (PR), increasing the R0 resection rate, improving the overall survival (OS) and disease-free survival (DFS), have been verified by numerous large-scale RCTs (6–8). In 2007, NACT was officially adopted by NCCN guidelines, and it has become the standard treatment for LAGC patients since then (5).

However, due to the tumor heterogeneity, the clinical response rate of NACT is barely satisfactory (9). For patients who are resistant to NACT, the operation timing is postponed while the primary tumor may progress during the treatment. More than that, the NACT-induced adverse events which could have been avoided, may deteriorate the general condition of those patients (10, 11). Therefore, mining the reliable indicators that can predict the sensitivity of chemotherapy is in great need, so as to screen out GC patients who are suitable for NACT. In addition, the optimal course of NACT is still uncertain, so monitoring indicators can help to evaluate the efficacy of NACT and select the appropriate surgical timing in real time. All of the above are hot topics which can boost the effect of NACT and help achieve the individualized treatments for GC patients.

In this article, we reviewed six categories of indicators which showed promising effects in predicting and monitoring the sensitivity and efficacy of NACT in GC treatment. Although most of them have not been studied thoroughly and few conclusions have been drawn, it will inspire us to design a well-organized and individual-oriented NACT strategy for GC patients.

## miRNA

MicroRNA (miRNA) is a type of non-coding single-stranded small RNA molecule with a length of about 18–25 nucleotides, and more than 900 miRNAs have been identified (12). By binding to the 3 ′UTRs of mRNAs, miRNAs regulate the expression of more than one-third of genes and participate in varies biological processes (13). Recently, studies have shown that the abundance of some miRNAs may help to explain the mechanisms of chemotherapy; thus, the variation of them can be used for chemoresistance monitoring (**Table 1**).

**Table 1:** The potential mechanism of miRNAs for causing chemotherapy resistance in GC.

Mechanism		miRNAs	Target(s)	Expression	Drug
**Apoptosis**	**BCL-2**	miR-15b, miR-16, miR-200bc/429	BCL-2	↓	DDP,ADR, VCR,VP-16
		miR-497			DDP, ADR, VP-16
		miR-1217, miR-143, miR-136			DDP
		miR-429			5-FU
		miR-181b			5-FU,DDP, ADR,VCR, VP-16
		miR-204			5-FU,OXA
	**PI3K/Akt**	miR-193-3p, miR-147	PTEN	↑	5-FU
		miR-106a, miR-21-5p			DDP
		miR-4295	LRIGI		
		miR-375	ERBB2	↓	
		miR-126	EGFA		
		miR-19a/b	PTEN	↑	DDP, ADR,5-FU
		miR-316-5p	FOXMI	↓	Docetaxel
	**MAPK**	miR-206	MAPK	↓	DDP
		miR-135b-5p	MSTI, KLF4	↑	
	**NF-κB**	miR-145	APRIL	↓	DDP
		miR-20a	CYLD	↑	
	**Cell cycle arrest**	miR-31	ZH2	↓	5-FU
		miR-223	FBXW7	↑	DDP
**Elevated drug efflux**		miR-27a	BCL-2, P-gp, LRP	↓	OXA
		miR-508-5p	ABCBI, ZNRDI		5-FU,DDP, VCR,ADR
		miR-361-3p	ABCBI		OXA
		miR-21	P-gp	↑	PTX
		miR-19a/b			ADR
		miR-30a		↓	DDP
**Autophagy**			LC3-II		
		miR-181a	ATG5		
		miR-23b-3p	ATGI2		5-FU,DDP, VCR
		miR-148a-3p	AKAPI, RABI2		DDP
**Drug targets**		miR-34c-5p	MAPT	↓	PTX

ADR, doxorubicin; VCR, vincristine; Vp-16, etoposide; DDP, cisplatin; PTX, paclitaxel; 5-FU, fluorouracil; OXA, oxaliplatin.

Expression level: Upregulation(↑) or down-regulation(↓) of miRNA in drug-resistant GC cell lines compared with that in parental cells.

### Apoptosis

Drug induced endogenous and exogenous apoptosis in cancer cells is one of the main mechanisms of chemotherapy (14). However, this process can be impaired by miRNAs *via* regulating the expression of certain genes, which participate in apoptosis-related signaling pathways.

BCL-2 is one of the most important anti-apoptotic genes and is also frequently regulated by miRNAs. In 2008, Xia et al. (15) found that miR-15b and miR-16 were down-regulated in GC-resistant cell lines SGC7901/VCR. Meanwhile, over-expression of miR-15b and miR-16 can increase the apoptosis of normal GC cells (SGC7901) by inhibiting the expression of the BCL-2, thereby reducing the resistance to adriamycin (ADR), vincristine (VCR), etoposide (VP-16), and cisplatin (DDP). Later, more mechanisms have been proposed to explain the miRNA-mediated chemotherapy resistance of GC cells on BCL-2, such as miR-200bc/429, miR-1217, miR-143, and so on (16–18). These results partially elaborated the chemoresistance to DDP, 5-FU, *etc*, as well as the multidrug resistance (MDR).

In addition to BCL-2 protein, miRNA can also regulate the apoptosis of GC cells induced by chemotherapy drugs through other pathways. For example, miR-193-3p (19), miR-147 (20), miR-106a (21), miR-21-5p (22), and miR-19a /b (23) are all highly expressed in drug-resistant GC cell lines, and reducing their expression will inhibit the PI3K/Akt cellular signal transduction pathway of by promoting the expression of PTEN, thus promoting the apoptosis of GC cells. Additionally, up-regulating miR-206 (24) expression can weaken the proliferation of drug-resistant GC cells, facilitate cell apoptosis, and decrease DDP resistance *via* targeted inhibition of MAPK3 (mitogen activating protein kinase 3) expression. The down-regulation of miR-135b-5p (25) induced apoptosis, and it inhibited proliferation and DDP resistance of GC cells by inactivating the MAPK signaling pathway and increasing the expression of MST1 (mammalian ste20-like kinase 1). The canonical NF-*κ*B pathway was involved in DDP resistance too. For example, miR-145 (26) regulated the sensitivity of GC cells to DDP by regulating the expression of APRIL (a proliferation-inducing ligand) through NF-*κ*B pathway. MiR-20a (27) directly repressed the expression of CYLD (cylindromatosis), leading to activation of the NF-*κ*B pathway and the downstream targets, livin and survivin (members of the inhibitor of apoptosis protein family, function as anti-apoptotic factors), which potentially induced GC chemoresistance. Moreover, miR-31 (28) and miR-223 (29) lead to apoptosis by blocking the cell cycle in the DNA replication process, thus enhancing the chemotherapy sensitivity of 5-FU and DDP for GC.

### Elevated Drug Efflux

Chemotherapy resistance in GC is also associated with drug efflux caused by the overexpression of some membrane transporters, the most important of which is the ATP-binding cassette (ABC) transporter family (30, 31), which is represented by P-glycoprotein (P-gp), which can pump anti-tumor drugs from inside to outside so that tumor cells can escape from the cytotoxic effect of and show resistance to chemotherapy drugs (30).

Some studies have conducted in-depth studies on the relationship between miRNA and GC chemotherapy resistance from the perspective of P-gp pathway. A study published by Zhao (32) in 2011 showed that down-regulation of miR-27a could significantly reduce the expression of P-gp and decrease the transport of ADR, leading to the accumulation of ADR in GC cells, thus enhancing the sensitivity of chemotherapy. Further studies by Zhao (33) in 2015 showed that hypoxia-inducible factor (HIF)-1 radiation influenced the expression of P-gp, LRP, and BCL-2 by regulating miR-27a, resulting in chemotherapy resistance of GC cells. In addition, miR-19a/b (23), miR-508-5p (34), miR-30a (35), miR-21 (36) and miR-361-3p (37) have also been proved to increase the excretion of chemotherapy drugs by regulating the expression of P-gp on the membranes of GC cells, resulting in decreased sensitivity to chemotherapy.

### Other Pathways for GC Chemo-Resistance

The mechanism of GC chemotherapy resistance is very complicated. In addition to the abovementioned apoptosis, cell cycle changes, and efflux of chemotherapy drugs, there are other mechanisms involved, such as autophagy and changes in drug targets.

In normal cells, autophagy can play an anticancer role by maintaining gene stability, while in cancer cells, autophagy can provide energy to cancer cells and promote the survival of tumor cells under stressful conditions, such as radiotherapy or chemotherapy (38). MiRNAs can also regulate the autophagy of tumor cells, causing drug resistance to NACT. For example, miR-30a (39), miR-181a (40), and miR-148a-3p (41) were confirmed to have low expression in drug-resistant GC cells, and *in vivo* and *in vitro* experiments showed that they all caused DDP resistance by regulating the autophagy of GC cells. Additionally, miR-23b-3p (42) leads to enhanced autophagy of GC cells through targeted regulation of ATGI2 (autophagy-related gene 12), thereby causing drug resistance to DDP, 5-FU, and VCR.

The changes in the chemotherapy drug targets caused by miRNA in GC have also been investigated. In 2013, Wu (43) reported that mir-34c-5p can regulate the expression of microtubule-associated protein tau (MAPT), which can stabilize the microtubule structure by promoting the accumulation of tubule proteins into microtubules. They found that the decreased expression of miR-34c-5p in paclitaxel-resistant GC tissues was accompanied by the increase of MAPT levels. Upon regulation of miR-34c-5p, the expression of MAPT was significantly reduced, leading to the increased sensitivity of drug-resistant GC cells to paclitaxel (PTX).

MicroRNA plays important roles in cell development, proliferation, differentiation, apoptosis, gene regulation, and disease occurrence, especially in the development of tumors and drug resistance. Changing the expression level of miRNA in tumors is expected to become a new treatment strategy. With the deepening of research, more and more miRNAs will become molecular markers to judge the sensitivity and prognosis of tumor treatment, guide individualized treatment, and improve the tumor therapeutic effect.

## Metabolic Enzymes Related to Fluorouracil Resistance

Regarding NACT for GC, NCCN guidelines have been updated continuously in recent years. Fluorouracil and cisplatin (FP) were classified as a category 1 recommendation in 2013, and fluorouracil and oxaliplatin were classified as a category 2A recommendation in 2017. In 2018, docetaxel, oxaliplatin, leucovorin, and fluorouracil (FLOT) were listed as a category 1 recommendation for NACT of GC (5). Therefore, fluorouracil is always used in NACT for GC, but due to its drug resistance, the single-drug effective rate of 5-FU is only 20%, and the overall effective rate of the first-line chemotherapy based on 5-FU is less than 40%; thus some patients cannot benefit from NACT (44). Therefore, it has become an urgent problem to explore the indicators related to the sensitivity of 5-FU drugs to chemotherapy. Among them, thymidylate synthase (TS), thymidine phosphorylase (TP) and dihydropyrimidine dehydrogenase (DPD) are the research hotspots.

5-FU is a thynoside synthase inhibitor. When 5-FU penetrates tumor cells, it is converted into fluorouracil deoxynucleotide (Fd UMP), which is covalently combined with reduced tetrahydrofolic acid (CH2FH4) and TS, forming a ternary complex to inhibit the activity of TS and interfere with DNA synthesis of tumor cells (45). TP is the last rate-limiting enzyme for the conversion of 5-FU prodrugs to fluorouracil. After oral administration of the drug into the body, 5-FU is converted into 5-fluoro-2-deoxyuracil nucleotide (Fd Urd) in the liver. On the one hand, the drug is converted into Fd UMP to inhibit TS. On the other hand, the drug is catalyzed into 5-FU by TP (46). DPD is the starting and rate-limiting enzyme of 5-FU catabolism. More than 85% of 5-FU is reduced into inactive metabolites by DPD in liver and other tissues, which are excreted by the kidney; thus, the activity of DPD is closely related to the efficiency of 5-FU (47). Therefore, the relationship between the expression levels of the enzymes TS, TP, and DPD and the chemotherapy sensitivity and prognosis of patients with GC to 5-FU is still a hot topic in the field of NACT.

Initially, in 2000, Salonga (48) found that colon cancer patients with low expression levels of DPD, TS, and TP before chemotherapy were sensitive to 5-FU. Subsequently, in 2002, Tershima (49) reported that the activity of DPD in GC tissues could predict the chemotherapy sensitivity and drug resistance of tumors to 5-FU. In 2004, Wang (50) detected overexpressed TS in the analysis of 5-FU-resistant cancer cell lines by DNA microarray, and Etienne (51) also confirmed that TS was closely related to the chemotherapy sensitivity of 5-FU. In the same year, Ma (52) evaluated four kinds of GC cells and three kinds of colon cancer cells and found that cell lines with low DPD expression level were more sensitive to 5-FU, and DPD mRNA could not even be detected in the most sensitive cell line HCT-8. At the same time, it was found that TS may contribute greatly to the sensitivity of FdUrd, and the higher the TS mRNA levels, the higher the IC50 (50% growth inhibitory concentration) of FdUrd. Then Napieralski (53) found that patients with high DPD expression were insensitive to 5-FU and had poor prognosis, while patients with low DPD expression were just the opposite. Sasako M (54) showed that high TS and DPD gene expression in tumors was associated with enhanced benefit from postoperative adjuvant S-1 treatment in gastric cancer. Later in 2016, a meta-analysis covered 555 patients with GC treated with S-1 showed that there was a significant difference in ORR (objective response rate) between patients with high/+ and low/− expression of DPD (55).

It can be seen from the above studies that the enzymes related to fluorouracil metabolism have always been the focus of NACT, but they are mainly limited to *in vitro* experiments; thus, the systematic *in vivo* experiments on NACT of GC still require further exploration.

## Exosomes

Exosomes are also one of the star molecules in new biomarkers. They are small lipid bilayer extracellular vesicles loading a variety of cargo, including DNA, mRNA, miRNA, circular RNA, protein, *etc* (56), typically 30–100 nm in size, and can be detected in various biological fluids, such as serum, urine and saliva (57). More and more reports have shown that exosomes play an important role in tumor growth, metastasis, angiogenesis and immune regulation by acting as information communicators (58–60). Moreover, exosomes have recently been found to be involved in the regulation of cancer chemoresistance (61–63), GC is surely included.

In 2020 Sun MY (64) demonstrated that RPS3 (Ribosomal Protein S3) expression levels were significantly elevated in cisplatin-resistant gastric cancer cell line SGC7901 and the exosomal delivery of RPS3 might induce chemoresistance phenotypes from cisplatin-resistant gastric cancer cells to sensitive cancer cells by regulating the PI3K-Akt-cofilin-1 signaling pathway. And Zhang QM (65) indicated that exosomes with si-c-Met can inhibit the invasion and migration of GC cells and promote apoptosis *in vitro* and inhibit tumor growth *in vivo*, reversing the resistance to cisplatin in GC.

In addition to proteins, RNAs in exosomes were also found to be associated with chemosensitivity in GC. Zhang HY (66) showed that cisplatin and paclitaxel promoted exosomal miR-522 secretion from CAFs (cancer-associated fibroblasts), leading to ALOX15 (arachidonate lipoxygenase 15) suppression and decreased lipid-ROS (toxic lipid peroxides) accumulation in GC cells, and ultimately result in decreased chemo-sensitivity. Furthermore, it was reported that exosome miR-155-5p directly inhibits GATA3 (GATA binding protein 3) and TP53INP1 (tumor protein p53-induced nuclear protein 1) to induce paclitaxel resistant GC cells to sensitive ones (67). And Wang SM (68) found that exosomal circPRRX1 (circular paired-related homeobox 1) strengthened doxorubicin resistance of GC cells by modulating miR-3064-5p/PTPN14 (non-receptor tyrosine phosphatase 14) signaling pathway.

Exosomes contain not only proteins but also a significant amount of nucleic acids, including DNA, mRNAs, miRNAs, circular RNAs (circRNAs) and long non-coding RNAs (lncRNAs) (69), as well as cholesterols, diglycerides, phospholipids, glycerophospholipids, sphingomyelins, and ceramides (70). In order to identify more sensitive and specific exosomes to guide individual chemotherapy choices, future studies should further clarify the roles and potential mechanisms of exosomes in cancer with more chemotherapeutic drugs.

## Other Chemotherapy-Related Biomarkers

Biomarkers are the most cutting-edge research direction for the prediction of the chemotherapy sensitivity of GC. In addition to miRNAs and fluorouracil metabolic enzymes, glutathione S-transferase (GST) is also involved. In 2006, Goekkurt (71) analyzed the polymorphism of GST genes in 52 patients with LAGC who received 5-Fu or DDP in NACT and found that the efficacy in patients with the GSTP-105Val/Val gene subtype (67%) was better than those with at least one 105Ile allele (21%).

In 2017, Li (72) detected the expression of P-glycoprotein (P-gp), glutathione S-transferase-*π* (GST-*π*), topoisomerase II (topo II), multidrug resistance gene-associated protein (MRP), lung resistance-related protein (LRP), Ki-67, and p53 in cancer tissues of 93 elderly patients with AGEJ (adenocarcinoma of gastroesophageal junction) before NACT and then analyzed the relationship between the expression of these proteins and the curative effect of NACT. The results showed that only the expressions levels of ki-67 (p = 0.003) and p53 (p = 0.009) were significantly correlated with the sensitivity to NACT, and the increased expression of ki-67 and the decreased expression of p53 predicted the SOX insensitivity of elderly patients with AGEJ.

In 2019, Hashimoto (73) compared the mismatch repair genes MLH-1 and PD-L1 of 110 GC patients who received different NACTs with 175 patients who did not receive any NACT and found that the NACT response of MLH1-negative patients was significantly lower than that of MLH1-positive patients (16.7 *vs.* 61.2%, P = 0.005), while there was no significant difference between patients with high and low PD-L1 expression (55.9 *vs.* 56.6%, P = 0.95). Therefore, it is recommended that MLH1-negative patients with GC should be treated with surgery alone, while patients with other types of GC should be treated with a combination of surgery and preoperative or postoperative chemotherapy. The study also showed that poor prognosis of MLH1-positive patients with GC can be improved by NACT. At the same time, PD-L1 expression did not have any predictive characteristics for prognosis or NACT response.

With the characterization of more biomarkers and the improvement of various detection levels, a growing number of markers such as tumor markers (for example, CEA, CA19-9, CA153, CA72-4, AFP) (74), circulating free DNA (cfDNA) (75), and circulating tumor cells (CTC) (76), were used to predict the sensitivity of NACT for GC to provide clinical evidence of individualized diagnoses and treatment plans. However, due to the small sample size, different chemotherapy options, and the presence of so many biomarkers, but very few with specificities, the use of biomarkers to specifically predict the sensitivity of NACT for GC still requires our further efforts and exploration.

## Inflammatory Markers

Some studies have found that the occurrence and development of tumors are closely related to systemic inflammatory responses (77), and some of the inflammatory markers may be associated with the effectiveness of NACT (78). A 2014 study by Borsig (79) noted that peripheral blood tests can reflect the level of inflammation at the time of tumorigenesis and that inflammatory markers such as C-reactive protein (CRP), white blood cells (WBC), and neutrophil–lymphocyte ratio (NLR) and platelet–lymphocyte ratio (PLR) can all be used as prognostic factors for patients with various malignancies. In LAGC, high NLR is considered to be an effective predictor of survival, and in 2014, Mohamed (80) found that a high level of NLR indicates a worse PFS (progression free survival) and OS (overall survival) in patients with LAGC undergoing NACT. The Glasgow prognostic score (GPS), which is calculated from CRP and serum albumin (ALB), is considered as a comprehensive indicator reflecting the systemic inflammatory response and nutritional status. Studies have shown that in some tumors, GPS is related to the effectiveness of NACT and prognosis. In patients with AGEJ, an increase in GPS score may indicate a decrease in the tolerance and efficacy of NACT and a reduction in survival time.

In the tumor microenvironment, macrophages are known as tumor-associated macrophages (TAMs), which are one of the most abundant immune cells. The degree of TAMs’ infiltration in tumor tissues was positively correlated with the adverse prognosis of various tumors, including GC. TAMs promote tumor progression by secreting a variety of inflammatory factors, including growth factors, chemokines, and cytokines (81). Macrophages are divided into M1 and M2 types. M1 macrophages have pro-inflammatory effects, producing various cytokines and chemokines, such as IL-12 (interleukin 12), CXCL9 (C-X-C motif ligand 9), and TNF-α (tumor necrosis factor-α) (82). M2 macrophages produce anti-inflammatory cytokines, such as TGF-*β* (transforming growth factor-*β*) and IL-10 (83). In the GC mouse models, macrophages were recruited by chemokines and cytokines derived by epithelia (84–89), and they produced pro-inflammatory cytokines such as TNF-α and stimulated tumor growth (88, 90). Moreover, the depletion of macrophages in these mouse models inhibited the proliferation and tumorigenesis of epithelia (87, 89, 90). Besides, gene expression and a novel associated cytokine panel were also linked to GC metastasis. For example, in 2020 Qeadan (91) found that MK2 (Map kinase-activated protein kinase 2) expression and a panel of associated cytokines secreted by GC cells, including G-CSF (granulocyte colony-stimulating factor), GM-CSF (granulocyte-macrophage colony-stimulating factor), Mip-1*β* (macrophage inflammatory protein-1*β*), IFN-*α* (interferon-*α*), MCP-1 (monocyte chemotactic protein 1), IL-1*β*, IL-6, and TNF-*α* to be linked to GC metastasis. But more future studies are needed to clarify the precise role of macrophages, cytokines, and other inflammatory markers in the NACT of GC.

## CT, MRI, PET and Other Imaging Evaluation Indicators

At present, the evaluation of the efficacy of chemotherapy for GC is still mainly based on the response evaluation criteria in solid tumors (RECIST) and WHO (World Health Organization) standards, which are evaluated by measuring the changes of maximum diameter and area of tumors before and after chemotherapy (92, 93). However, the stomach is a cavity organ, thus changes in its wall thickness, peristalsis, tumor morphology, and measurement angle will make the measurements of lesion size inaccurate (94). Theoretically, the morphological changes are the result of changes in the biological behavior of the lesion. After NACT, the lesions should have already changed functionally before morphological changes occurred, mainly manifesting as the reduction of tumor local blood perfusion (95). Therefore, if imageological assessments can be used to observe tumor-associated vessels in GC patients, it would be of great clinical significance.

In 2014, Hansen found that after chemotherapy for GC, the tumor volume and surface permeability value (an indicator of tumor local vascular permeability) in CT (computed tomography) perfusion parameters both decreased significantly (96). Moreover, energy spectral CT can accurately reflect the true iodine concentration (97), and iodine concentration can accurately reflect the blood supply and vascular conditions of lesions (98), so the energy spectrum CT is also used to evaluate the vascularization after NACT for GC. In 2005, Tang’s study showed that there was a significant difference in iodine concentration in the arteriovenous phase before and after NACT, and the change of iodine concentration was significantly correlated with tumor regression grade (99). Some studies also used iodine uptake (IU) as a functional parameter to assess the sensitivity of chemotherapy in other tumors, but further research is still needed on NACT for GC. There are also other imageological assessments. For instance, in 2016, Lee analyzed 11 LAGC patients after NACT by PET and MRI(magnetic resonance imaging), showing that K (trans) and iAUC (initial area under the curves) values can be used as early predictive markers for chemotherapy response (100). Latter in 2018, the study of Schneider showed that in GC or AGEJ patients, after the first cycle of NACT PET-CT (positron emission tomography–computed tomography)cannot accurately predict the overall pathological response, but it can accurately detect patients who are insensitive to NACT and should be operated upon immediately or treated in combination with other methods (101).

## Conclusion

NACT is currently an effective treatment for GC, but not all patients are sensitive to it. Therefore, searching for specific indicators to predict the sensitivity of NACT in GC, individualized diagnosis and treatment are still important parts of clinical research content. At present, whether biomarkers, inflammatory markers, or imageological assessments are used, it is still difficult to select patients who are sensitive to NACT. Most of the current research involves single-center macroscopic studies. Therefore, multicenter studies with larger samples in terms of proteomics, transcriptomics, and genomics, are needed to select the indicators for predicting the efficacy and prognosis of NACT to help screen out patients for tailored treatments.

## Author Contributions

JS and XW were responsible for collecting, sorting out data and writing articles, were co-first authors. ZZh, ZZe and SO were responsible for collecting data. WK was responsible for putting forward ideas and reviewing articles, was corresponding author of this paper. All authors approved it for publication. All authors contributed to the article and approved the submitted version.

## Funding

CSCO-ROCHE Research Fund No. Y-2019 Roche-015; Beijing Xisike Clinical Oncology Research Foundation Y-HS2019-43; Wu Jieping Medical Foundation No. 320. 6750.19020

## Conflict of Interest

The authors declare that the research was conducted in the absence of any commercial or financial relationships that could be construed as a potential conflict of interest.
